# Supplementary material for: Content-rich biological network constructed by mining PubMed abstracts
Source: BMC Bioinformatics. 2004 Oct 8;5:147. doi: 10.1186/1471-2105-5-147 (PMC528731; doi:10.1186/1471-2105-5-147)
Supplement: Additional File 5 — The original Chilibot query results of the term "long-term potentiation (LTP)" and 22 other terms, limiting the latest references analyzed to the years 1990, 1995, 2000, and 2004. [file 1471-2105-5-147-S5.bz2 › chilibotAdditionalFile5/ltp2004/html/AMPA_CAMKIV.html]

 


 **AMPA** and **CAMKIV** 
  
Found 1 abstracts in PubMed,  **1 abstracts were retrieved and analyzed**.  


---

 Search Google  |
 PDF files only 
|  EDU domain only 

---

- Neuron, 2003   **Calcium channel and NMDA receptor activities differentially regulate nuclear C EBPbeta levels to control neuronal survival.** .
  Insulin like growth factor 1 IGF 1 promotes the survival of cerebellar granule neurons by enhancing calcium influx through L type calcium channels.
  . . . NMDA receptor mediated calcium influx can lead to excitotoxic death.
  Here we demonstrate that L and NMDA receptor channel activities differentially regulate the transcription factor C EBPbeta to control neuronal survival.
  Specifically, we show that L channel dependent calcium influx results in increased **CaMKIV** activity, which acts to decrease nuclear C EBPbeta levels.
  Conversely, NMDA receptor mediated influx rapidly elevates nuclear C EBPbeta and induces excitotoxic death via activation of the calcium dependent phosphatase, calcineurin.
  Moderate levels of **AMPA** receptor activity stimulate L channels to improve survival.
  . . . higher levels stimulate NMDA receptors and reduce neuronal survival, suggesting differential synaptic effects.
  Finally, N type calcium channel activity reduces survival, potentially by increasing glutamate release.
  Together, these results show that the L type calcium channel dependent survival and NMDA receptor death pathways converge to regulate nuclear C EBPbeta levels, which appears to be pivotal in these mechanisms.
